# Spatiotemporal dynamics of Puumala hantavirus associated with its rodent host, *Myodes glareolus*

**DOI:** 10.1111/eva.12263

**Published:** 2015-05-29

**Authors:** Vanessa Weber de Melo, Hanan Sheikh Ali, Jona Freise, Denise Kühnert, Sandra Essbauer, Marc Mertens, Konrad M Wanka, Stephan Drewes, Rainer G Ulrich, Gerald Heckel

**Affiliations:** 1 Computational and Molecular Population Genetics (CMPG), Institute of Ecology and Evolution, University of BernBern, Switzerland; 2 Institute for Novel and Emerging Infectious Diseases, Friedrich-Loeffler-Institut, Federal Research Institute for Animal HealthGreifswald-Insel Riems, Germany; 3 College of Veterinary Medicine, Sudan University of Science and TechnologyKhartoum, Sudan; 4 Fachbereich Schädlingsbekämpfung, Niedersächsisches Landesamt für Verbraucherschutz und LebensmittelsicherheitWardenburg, Germany; 5 Department of Environmental Systems Science, Eidgenössische Technische Hochschule ZürichZürich, Switzerland; 6 Department of Virology & Rickettsiology, Bundeswehr Institute of MicrobiologyMunich, Germany; 7 Swiss Institute of BioinformaticsLausanne, Switzerland

**Keywords:** bank vole, genetic structure, hantavirus, host–parasite evolution, nephropathia epidemica, population dynamics, rodent-borne disease, zoonosis

## Abstract

Many viruses significantly impact human and animal health. Understanding the population dynamics of these viruses and their hosts can provide important insights for epidemiology and virus evolution. Puumala virus (PUUV) is a European hantavirus that may cause regional outbreaks of hemorrhagic fever with renal syndrome in humans. Here, we analyzed the spatiotemporal dynamics of PUUV circulating in local populations of its rodent reservoir host, the bank vole (*Myodes glareolus*) during eight years. Phylogenetic and population genetic analyses of all three genome segments of PUUV showed strong geographical structuring at a very local scale. There was a high temporal turnover of virus strains in the local bank vole populations, but several virus strains persisted through multiple years. Phylodynamic analyses showed no significant changes in the local effective population sizes of PUUV, although vole numbers and virus prevalence fluctuated widely. Microsatellite data demonstrated also a temporally persisting subdivision between local vole populations, but these groups did not correspond to the subdivision in the virus strains. We conclude that restricted transmission between vole populations and genetic drift play important roles in shaping the genetic structure and temporal dynamics of PUUV in its natural host which has several implications for zoonotic risks of the human population.

## Introduction

The evolution of pathogens is profoundly influenced by the evolutionary history and geographical distribution of their hosts. Connectivity between host populations and their local sizes determine to a large extent the transmission and infection rates and geographical distribution of a pathogen. However, pathogens may evolve specific strategies or use windows of opportunity to overcome the limitations of particular host species, resulting in host-switch events and the (re-)emergence of infectious diseases (e.g., malaria, influenza, AIDS, and Ebola). Emerging infectious diseases may thus be examples where pathogens escape the evolutionary constraints of particular hosts and enter different evolutionary backgrounds (Cox-Singh 2012; Morse et al. 2012).

The number of emerging infectious diseases is increasing, and these pathogens have a profound impact on public and animal health as well as on the economy (Jones et al. 2008). A large proportion of the emerging diseases represent zoonoses caused by RNA viruses that are transmitted to humans from their natural reservoir species (Cleaveland et al. 2001; Holmes 2009). Understanding the population dynamics and interactions between viruses and their natural hosts is essential for resolving epidemiological processes and their evolutionary trajectories (Gire et al. 2014).

Hantaviruses, segmented negative stranded RNA viruses from the *Bunyaviridae* family, are among the most important emerging infectious pathogens with often enigmatic epidemiology and transmission pathways (Vaheri et al. 2013b). In the Americas, they are responsible for hantavirus cardiopulmonary syndrome with relatively high case fatality rate in humans. Hantaviruses in Asia, Europe, and most likely also Africa can cause hemorrhagic fever with renal syndrome (HFRS) (Vaheri et al. 2013a). The natural hosts of hantaviruses are mostly small rodents (families Muridae and Cricetidae), but also insectivores from the order Soricomorpha (families Talpidae and Soricidae), and bats (Guo et al. 2013; Schlegel et al. 2014). To date, only hantaviruses harbored by rodents have been identified as pathogenic to humans. Transmission of the viruses to humans occurs mainly through inhalation of virus-contaminated aerosols of excreta from infected rodents (Vaheri et al. 2013a).

Puumala virus (PUUV) is a hantavirus that causes a mild to moderate form of HFRS in humans. The natural reservoir host of PUUV is the bank vole, *Myodes glareolus*, a small rodent species that occupies forested and wooded areas throughout most of Europe. PUUV causes a chronic infection in bank voles and may somewhat reduce survival particularly in winter (Kallio et al. 2007; Tersago et al. 2012). The prevalence of PUUV in bank vole populations ranges from absent to very high depending on the region of Europe, the local population, and the year (Razzauti et al. 2013). It has been suggested that PUUV outbreaks in humans are associated with high densities of bank voles caused by high tree seed production in the preceding year, but there is considerable geographical variation in these associations (Olsson et al. 2003; Kallio et al. 2009; Tersago et al. 2009, 2011).

Phylogenetic analyses of PUUV typically detect differences between sequences even at relatively small geographical distances (Escutenaire et al. 2001; Sironen et al. 2002; Razzauti et al. 2008; Mertens et al. 2011). This may be explained by the very high evolutionary rates of these RNA viruses, leading to divergent strains at different localities over short periods and/or the lack of effective transmission between bank vole populations. In central Europe, bank voles belong generally to the same phylogeographic lineage with no or very little substructure in mitochondrial DNA (Wójcik et al. 2010; Mertens et al. 2011). Autosomal markers can resolve population structures in bank voles at larger geographical scales (White et al. 2013), but populations may undergo very specific processes at the local level, for example, source–sink dynamics (Guivier et al. 2011) perturbing spatial patterns. Investigations directly linking the genetic structure of host populations with the genetic structure and molecular diversity of PUUV (or any other hantavirus) populations are lacking to date.

In this study, the following hypotheses were investigated: (i) Genetic substructure in Puumala virus populations reflects potential substructure in the host; (ii) the PUUV strains in local rodent populations change rapidly through time due to their high evolutionary rates and/or genetic drift; and (iii) temporal dynamics of PUUV in natural hosts and in human populations are related. To test these hypotheses, we combine population genetic analyses of PUUV and its natural host in an endemic area in central Europe. Molecular data from bank vole populations collected over eight years spanning several disease outbreaks in human populations in the region together with sequence information from all three segments of the virus genome provide in unprecedented detail insight into the processes of PUUV persistence and microevolution in its natural rodent host. Such information is pivotal for the establishment of targeted risk assessment and prevention measures for the human population particularly in PUUV endemic areas.

## Materials and methods

### Ethics statement

Rodent trapping was performed by the Lower Saxony State Office for Consumer Protection and Food Safety (JF) as part of the pest control measures against bank voles implemented and authorized by the health authorities of the district Osnabrück (https://www.landkreis-osnabrueck.de/veterinaer-gesundheit/infektionsschutz/mrsa) according to German federal law (§ 18, Gesetz zur Verhütung und Bekämpfung von Infektionskrankheiten beim Menschen). These measures were implemented after ethical review by the commission for infection protection of the district Osnabrück after repeated hantavirus infections in the human population in the district following the recommendations of the Robert Koch-Institut for disease control (www.rki.de; latest version: 10/2010).

### Sampling procedure and sample preservation

**Figure 1:**
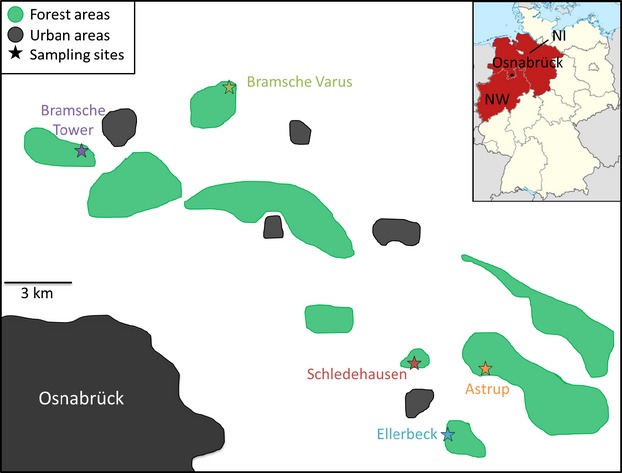
Map of the study region showing the five localities where bank voles (*Myodes glareolus*) were trapped between 2005 and 2012. Habitat features other than intensively used agricultural land that could be relevant for the species (forests and urban areas) are indicated. The inset shows the position of the study region in Germany, with the encompassing federal states Niedersachsen (NI) and Nordrhein-Westfalen (NW) highlighted.

Beginning in 2005, voles were trapped in a PUUV endemic area using mouse snap traps baited with raisins as part of a pest control and wildlife disease monitoring program. The five trapping sites in the district Osnabrück in northwestern Germany (Fig.1) can be characterized as broad-leaved forests dominated by the common beech (*Fagus sylvatica*) with scarce to no understory. After trapping, bank voles were immediately frozen. Carcasses were thawed overnight, and lungs and hearts were sampled under biosafety level 3 conditions. Chest cavity fluid (CCF) was collected by washing the chest cavity with 1 mL phosphate-buffered saline (Essbauer et al. 2006; Mertens et al. 2011), and samples were stored at −20°C until investigation.

### Serology, hantavirus RT-PCR, and sequencing

Serological screening of CCF was performed by IgG ELISA using yeast-expressed PUUV Vranica/Hällnäs and Bavaria nucleocapsid proteins (Essbauer et al. 2006; Mertens et al. 2011). RNA was extracted from lung and heart tissue using Qiazol solution (Qiagen, Hilden, Germany). The genome of PUUV consists of three segments, small (S), medium (M), and large (L), which encode the nucleocapsid protein, two surface glycoproteins, and a viral RNA-dependent RNA polymerase, respectively. An initial screening RT-PCR targeting the S segment was performed using a One Step protocol as described in Essbauer et al. (2006). The screening was repeated with modified primers (Table S1) for samples that were negative in the initial screen. Finally, a nested PCR protocol was used for the remaining negative samples (Essbauer et al. 2006). For S segment-positive samples, M- and L-segment-specific RT-PCRs were performed (Pilaski et al. 1994; Klempa et al. 2006; Mertens et al. 2011). For sequencing, amplicons were purified using QIAquick PCR purification kit according to the manufacturer's instructions (Qiagen). The products were sequenced at least three times using the BigDye terminator sequencing kit (Perkin-Elmer, Waltham, MA, USA) on an ABI 310 Genetic Analyzer (Applied Biosystems, Foster City, CA, USA).

### PUUV population structure and phylogenetic relationships

PUUV sequences were aligned manually using BioEdit 7.1.9 (Hall 1999). DnaSP version 5 (Librado and Rozas 2009) was used to determine the number of synonymous and nonsynonymous substitutions and to estimate nucleotide diversity in the PUUV sequences. Median-joining networks were produced with Network 4.6 (Bandelt et al. 1999), and genetic population structure was inferred with an analysis of molecular variance (amova) implemented in Arlequin 3.5 (Excoffier and Lischer 2010). Pairwise *F*_ST_ values were calculated for each PUUV segment separately and also for the three segments concatenated with 10 000 permutations to assess the level of statistical significance. BaTS (Parker et al. 2008) was used to determine the association between the phylogeny and the geographical location of the samples, by estimating the association index (AI), the parsimony score (PS) and the maximum clade (MC) size statistics. We tested for recombination and reassortment between the PUUV segments with RDP4 (Martin et al. 2010) analogous to the analyses in Fink et al. (2010).

Phylogenetic analyses were performed with the concatenated segments including four published PUUV sequences from the sampling sites (S segment: schle_05_001, varus_09_024, astrup_07_003; M segment: schle_05_015) and prototype strain Sotkamo as outgroup (accession numbers NC_005224, NC_005223, and NC_005225 for S, M, and L segments, respectively). Mega 5.1 (Tamura et al. 2011) was used to reconstruct phylogenetic trees based on neighbor-joining (NJ) algorithms. The HKY+G substitution model showed the best fit to our data based on the Bayesian Information Criterion tested in jModelTest 2.13 (Darriba et al. 2012). Bayesian phylogenetic analyses were performed with BEAST 1.7.5 (Drummond et al. 2012) on the Cipres portal (Miller et al. 2010). After initial tests, we used a strict molecular clock, a coalescent Bayesian skyline tree prior with 10 groups, and otherwise default priors for two runs of 100 million generations each with sampling every 20 000 generations. A burn-in of 10% was discarded, and convergence of model parameters was checked with Tracer 1.5 (Rambaut and Drummond 2007). The runs were combined using LogCombiner 1.7.5 (Drummond et al. 2012). A maximum clade credibility tree was produced with TreeAnnotator 1.7.5 and visualized in FigTree 1.4.0 (http://tree.bio.ed.ac.uk/software/figtree/).

### PUUV Bayesian skyline plot analyses

BEAST2 (Bouckaert et al. 2014) was used to shed light on the viral population dynamics. Bayesian skyline plot analysis (Drummond et al. 2005) was performed to estimate the effective population size and the substitution rate of PUUV based on the Schledehausen and Astrup datasets without the outgroup sequence. The two datasets were analyzed jointly, enabling the estimation of a common substitution rate. All other parameters, including the phylogenies, were estimated separately. The BEAST specifications remained as described above. The Bayesian skyline plots were drawn with Tracer. Path-O-Gen (http://tree.bio.ed.ac.uk/software/pathogen/) was used to regress the root-to-tip distance against the sampling date, in order to confirm the presence of temporal signal in the dataset.

### Bank vole multilocus genotyping

Genomic DNA was extracted from tail tissue using the phenol–chloroform method. The following 17 microsatellite loci were amplified in three sets, using the Qiagen Multiplex Kit in a PTC-100TM (MJ Research) thermocycler: CG1E6, CG1E8, CG2A4, CG5F6, CG5G6, CG6D10, CG7C9, CG12A7, CG12B9, CG13F9, CG13G2, CG15F7, CG16E2, CG16E5, CG17E9 (Rikalainen et al. 2008), MSCg-15 and MSCg-19 (Gockel et al. 1997). The amplification conditions were 95°C for 15 min, followed by 30 cycles of denaturation at 94°C for 30 s, annealing at 57°C for 90 s and extension at 72°C for 60 s, and 60°C for 30 min. Fragment separation was performed on an ABI 3130 sequencer. Fragment length was determined in comparison with the internal LIZ 500 size standard using GeneMapper 3.7 (Applied Biosystems). Repetitions of previously scored genotypes were performed to ensure genotyping consistency (Hahne et al. 2011).

### Vole population structure

Each vole sampling locality was checked for the presence of null alleles with MicroChecker 2.2 (Van Oosterhout et al. 2004). Deviations from Hardy-Weinberg equilibrium (HWE) were tested per population with Arlequin 3.5 (Excoffier and Lischer 2010). Pairwise *F*_ST_ between populations was computed as for the PUUV populations. We tested also for significant genetic changes in the bank vole populations over time, computing pairwise *F*_ST_ between samples from different years for the localities Schledehausen and Astrup, for which the largest sample sizes were available. Population structure in the voles was analyzed further with the clustering algorithm in Structure 2.3 (Pritchard et al. 2000), assuming an admixture model with correlated allele frequencies (Falush et al. 2003) and without information about the sampling population. We performed ten runs each for *K* between one and ten with 400 000 Markov chain Monte Carlo (MCMC) iterations and a burn-in of 40 000 iterations. The estimation of *K* followed the method suggested by Evanno et al. (2005), and the figures were displayed with Distruct 1.1 (Rosenberg 2004).

## Results

### Detection and genetic diversity of PUUV

**Figure 2:**
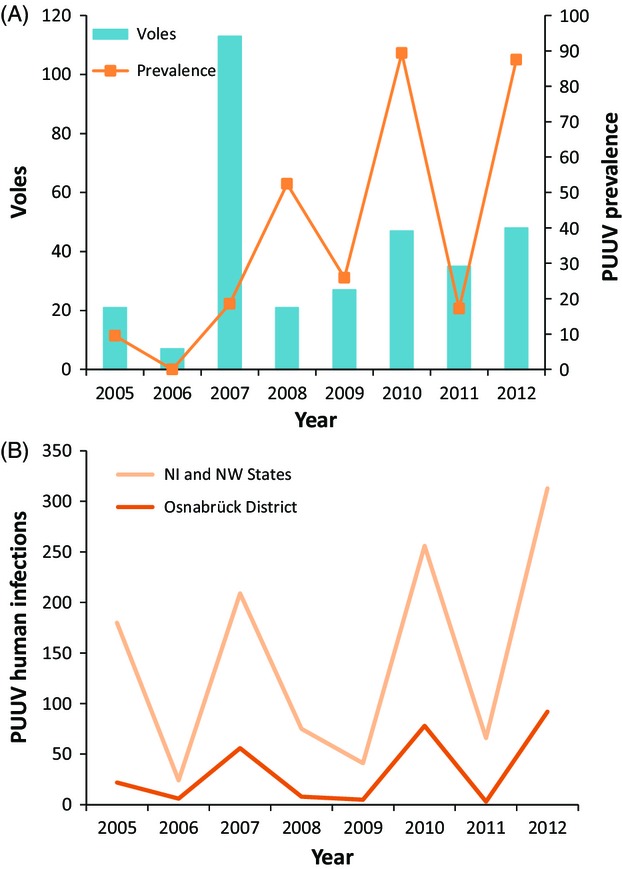
Puumala virus prevalence in local vole populations and human infections in the larger region. (A) Number of voles sampled and PUUV prevalence in each year for all sites in the study together. (B) PUUV human infections registered per year in the Osnabrück district and in the encompassing German federal states Niedersachsen (NI) and Nordrhein-Westfalen (NW) pooled together [data from Robert Koch-Institut (2013)].

We sampled 319 bank voles between 2005 and 2012 at five different localities with geographical distances between 2.5 and 17 km (Fig.1, Table1). PUUV-specific antibodies were detected in 128 voles (41%) by ELISA, and in three additional ones, PUUV RNA was detected only by RT-PCR (infected animals: total 131; 42%). There were large differences in the prevalence between bank vole populations and sampling years, ranging from 0% to 100% for localities with at least ten voles trapped per year and a maximum of 89% PUUV-infected voles (*n* = 47) across all localities in 2010 (Table1, Fig.2). Fluctuations in PUUV prevalence in the voles coincided only to some extent with the changes in the number of reported human infections in the Osnabrück district to which the localities belong administratively. Also the number of human infections reported in the German federal states Niedersachsen and Nordrhein-Westfalen encompassing the study district showed no strong association with local PUUV prevalence. For example, in 2007, human infections were frequently reported in the region (Fig.2), but PUUV prevalence was only moderate (19%) in the large number of voles sampled (*n* = 113).

**Table 1:** Overview of bank vole and Puumala virus samples used in this study

Locality	Year	Voles	Virus strains	S/M/L	S, M, L	Prevalence (%)
Schledehausen	2005	15	2	1/2/2	1	13.3
	2006	4	0	0	0	0^*^
	2007	37	5	4/4/4	4	13.5
	2008	7	4	3/3/3	3	57.1^*^
	2009	20	5	4/3/4	3	25
	2010	20	17	17/17/17	17	85
	2011	29	4	4/4/3	3	13.7
	2012	29	25	23/22/24	22	86.2
Astrup	2006	3	0	0	0	0^*^
	2007	28	10	9/10/10	9	35.7
	2008	12	6	5/5/6	5	50
	2009	3	1	1/1/1	1	33.3^*^
	2010	17	17	17/16/17	16	100
	2011	5	2	2/2/2	2	40^*^
	2012	15	13	12/12/11	10	86.66
Ellerbeck	2005	6	0	0	0	0^*^
	2007	18	5	5/5/5	5	27.7
	2008	1	1	1/1/1	1	100^*^
	2009	2	0	0	0	0^*^
	2010	6	5	5/5/5	5	83.3^*^
	2011	0	–	–	–	–
	2012	2	2	2/2/2	2	100^*^
Bramsche Varus	2007	22	0	0	0	0
	2008	1	0	0	0	0^*^
	2009	2	1	1/1/1	1	50^*^
	2010	4	3	3/3/3	3	75^*^
	2011	1	0	0	0	0^*^
	2012	2	2	2/1/2	1	100^*^
Bramsche Tower	2007	8	1	1/1/1	1	12.5^*^
	Total	319	131	122/120/124	115	41

Number of bank voles and Puumala virus (PUUV) strains at each trapping site per year, number of sequences for each PUUV genome segment (S/M/L) and the concatenated sequences (S, M, L), and PUUV prevalence in the bank voles. Asterisks indicate prevalence estimates based on vole sample sizes lower than 10.

Our sequences from the three PUUV segments S (637 nt), M (571 nt), and L (336 nt) cover in total about 13% of the entire genome. There was no indication of double peaks in the sequences potentially stemming from double infections or quasi-species. Nucleotide diversity per site varied from 0.0124 for the S, over 0.0162 for the M to 0.0196 for the L segment. There was a high diversity of virus sequences with 13 different types for the S segment, 20 for the M segment, 10 for the L segment, and 35 when all three segments were concatenated. Twenty-three synonymous and two nonsynonymous substitutions were found in the S segment, 30 synonymous and seven nonsynonymous substitutions in the M segment, and 16 synonymous and five nonsynonymous substitutions in the L segment. The highest number of substitutions between two sequence types was 16, 23, and 14 for the S, M, and L segments, respectively. Analyses with RDP4 (Martin et al. 2010) provided neither evidence of recombination between genome segments (=reassortment) nor of recombination within segments (all *P* > 0.05).

### Virus–geography relationships

PUUV populations from different localities were highly differentiated. For the concatenated virus sequences, overall population differentiation between localities was very high with *F*_ST_ = 0.25 (*P* < 0.001), a pattern mirrored in the segment-specific analyses (S: *F*_ST_ = 0.44; M: *F*_ST_ = 0.50; L: *F*_ST_ = 0.71; all *P* < 0.001). Pairwise *F*_ST_ values between populations ranged between 0.18 and 0.36 (all *P* ≤ 0.0011, except for the comparisons with Bramsche Tower; PUUV *n* = 1; Table S2). Again, the results of analyses of single segments matched the concatenated dataset with only slight quantitative and no qualitative differences (details not shown). Pairwise *F*_ST_ values for the PUUV samples from the same locality but from different years were also very high, but they were significantly different from zero only for some of the comparisons at the two localities with the largest population samples (Schledehausen and Astrup; Table S3). Association index and parsimony score from analyses with BaTS also confirmed the geographical clustering of PUUV sequences (*P* < 0.001), and maximum clade statistics revealed significant clustering in the sampling sites of Astrup, Ellerbeck, Schledehausen and Bramsche Varus (*P* < 0.01).

**Figure 3:**
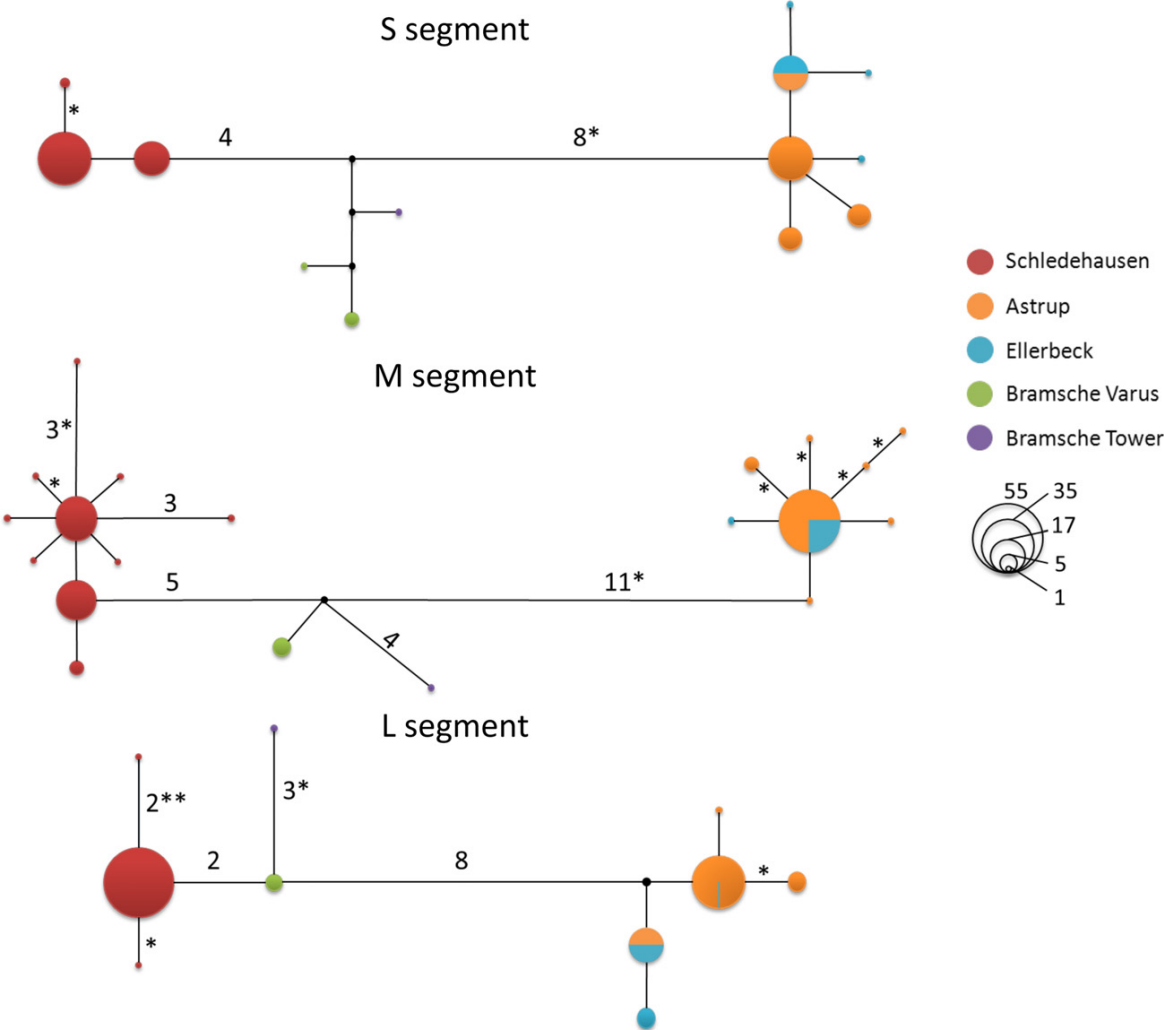
Median-joining networks based on S, M, and L segment sequences of Puumala virus strains obtained from bank voles (*Myodes glareolus*) sampled in the region of Osnabrück, Germany. Circles indicate PUUV sequence types, and colors indicate the trapping localities of *M. glareolus*. Circle sizes are proportional to the number of individuals sharing that sequence type. Numbers near the branches indicate how many substitutions separate the sequence types, and asterisks indicate the number of nonsynonymous substitutions. Branches without numbers represent one mutation.

In the haplotype network analyses, virus sequences always formed three clusters in the networks, each consisting of samples from the same localities—irrespective of the genome segment analyzed (Fig.3). The first cluster contains Schledehausen sequences only, the second cluster consists of Bramsche Tower and Bramsche Varus, and the third cluster is a composite of all sequences from Astrup and Ellerbeck. For each genome segment, at least one sequence type was shared between the Astrup and Ellerbeck localities, and even after concatenation of the segments resulting in 1544 nt, there was one sequence type that was found in several voles at both localities (Astrup *n* = 7; Ellerbeck *n* = 4, details not shown). In general, each geographical cluster contained one or two abundant sequence types and several closely connected rare ones, which differ from the abundant ones by one or two nonsynonymous substitutions at maximum (Fig.3). Interestingly, the number of substitutions separating the Astrup/Ellerbeck and Schledehausen sequences (geographical distance approx. 3 km) is largest for all segments, and viruses from the geographically distant Bramsche sampling sites (approx. 14 km distance) were genetically distinct but more similar to Schledehausen than to Astrup/Ellerbeck sequences.

**Figure 4:**
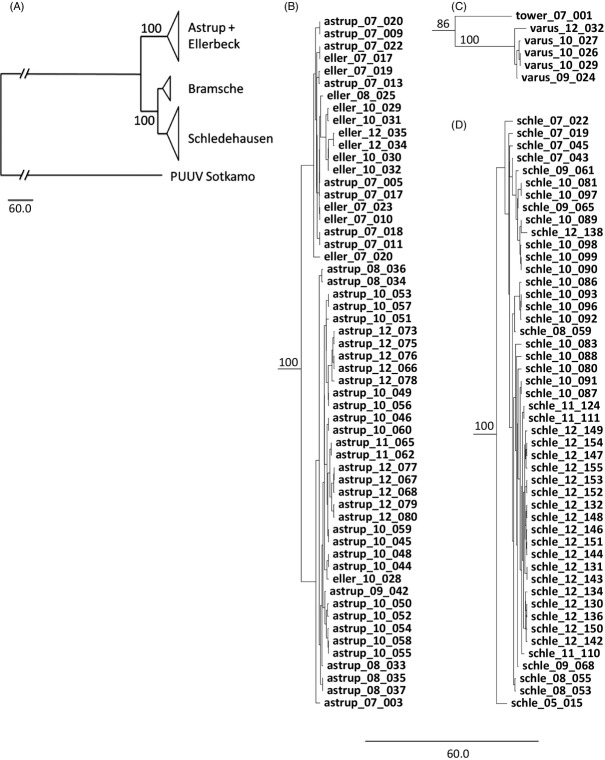
Coalescence-based, tip-dated phylogenetic tree inferred from the concatenated Puumala virus S, M, and L segment sequences with prototype strain Sotkamo as outgroup. (A) General topology of the phylogenetic tree of all analyzed virus sequences with condensed geographical clusters displayed in detail in B, C, and D. (B) Cluster formed by viruses from Astrup and Ellerbeck localities. (C) Cluster formed by viruses from the Bramsche locality. (D) Cluster formed by viruses from the Schledehausen locality. Sequence names indicate the geographical origin of the sample (schle, Schledehausen; astrup, Astrup; eller, Ellerbeck; varus, Bramsche Varus; tower, Bramsche Tower), followed by two digits indicating the sampling year. Posterior probabilities from Bayesian analyses are indicated close to the main branches.

Consistent with the networks, neighbor-joining and Bayesian phylogenetic analyses recovered the same three clusters in the concatenated sequences with very high support values (Fig.4, Figure S1). Also in these phylogenies, the Schledehausen and Bramsche virus clades were closer related to each other than to the clade that joined the PUUV sequences from the Astrup and Ellerbeck populations.

### Persistence of PUUV types through time

**Figure 5:**
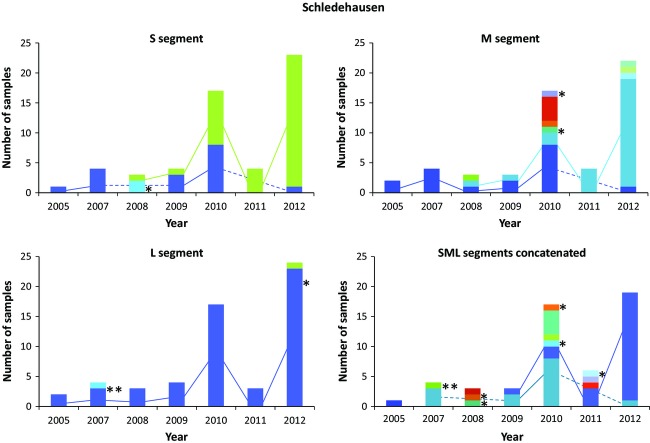
Persistence of Puumala virus types in the Schledehausen vole population for each genome segment and the three segments concatenated. The colors indicate different virus sequence types, and the lines connect the same virus type in different years. Asterisks indicate virus types with nonsynonymous substitutions. For analogous plots for the Astrup population, see Figure S2 in the Supplementary Material.

A high turnover of virus types between years was detected in each vole population (Fig.5, Figure S2), yet a few virus sequence types persisted during the eight years of the study even for the most variable M segment, despite the high evolutionary rate of hantaviruses. In our largest single-location sample, the Schledehausen population, three M segment sequence types persisted through multiple years—two of them for at least five years (Fig.5). For the S and L segments, the common sequence types generally persisted across multiple years although they were not always sampled. Even for the concatenated sequences with the highest resolution but the lowest sample size (*n* = 53), two types were repeatedly found in the vole population at Schledehausen in at least four different years. Novel PUUV sequences containing nonsynonymous changes were repeatedly detected in this vole population, but only one of them was detected in multiple years and none of them rose to high frequency (Fig.5). The Astrup population with appropriate samples sizes for these analyses (*n* = 43) presented very similar patterns of persistence of sequence types during multiple years (Figure S2).

### Population dynamics of PUUV

**Figure 6:**
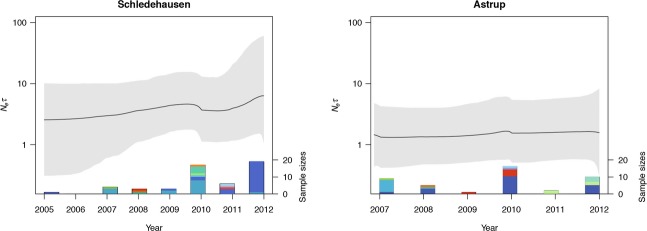
Bayesian skyline plots of effective population sizes of the Puumala virus populations Schledehausen and Astrup, based on concatenated S, M, and L segment sequences. The black line represents the mean estimate of the effective population size, and the gray area marks the highest posterior density interval. Bars on the *x*-axis indicate the number of virus sequences obtained at the respective locality in each year (right *y*-axis), and the colors indicate different virus sequence types.

Bayesian skyline plot analyses provided no evidence of significant changes in the effective population sizes of the Schledehausen and Astrup populations during the eight years of our study (Fig.6), despite large fluctuations in virus strain numbers and prevalence (Table1, Fig.2). For both populations, the 95% highest posterior density (HPD) intervals were relatively wide, and there was no obvious relationship between virus sample sizes and effective population size, consistent with the persistence of relatively few virus types over time. However, the large 95% HPD intervals suggest that the data do not contain enough information to estimate the effective population size very precisely. Analyses with Path-O-Gen revealed a significant correlation between root-to-tip tree distances and sampling time, confirming the suitability of this dataset for estimating substitution rates of PUUV. The median substitution rate of PUUV was estimated as 2.70 × 10^−4^ substitutions/site/year (95% HPD, 1.43 × 10^−4^–4.38 × 10^−4^).

### Genetic diversity of bank voles

The bank vole microsatellite loci presented between three and 47 alleles per locus and population, with expected heterozygosities ranging from 0.50 to 0.95 and observed heterozygosities from 0.24 to 1. Significant deviations from HWE were found at nine different loci in population-wise analyses, but this pattern was only consistent across the different populations for three loci. For these, we also detected the likely presence of null alleles with estimated frequencies of up to 0.37. However, performing all further analyses of vole data in parallel with all 17 loci or only with those that showed no consistent deviations from HWE or null alleles revealed only very minor quantitative and no qualitative differences (details not shown). We thus present in the following the results based on the full microsatellite dataset.

### Population structure of bank voles and PUUV

**Figure 7:**
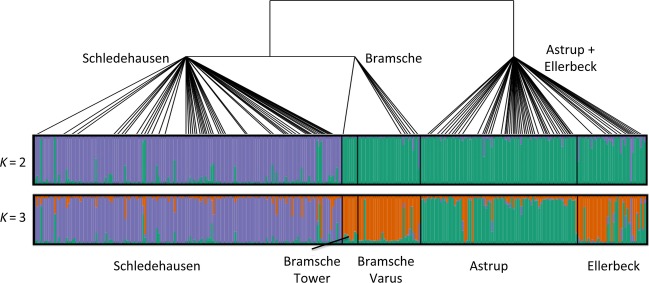
Genetic structure of local *Myodes glareolus* populations and schematic phylogenetic relationships of associated Puumala virus strains (see Fig.4). The first and second bars present the genetic structure of the bank vole populations based on 17 microsatellite loci computed with Structure for *K* = 2 and *K* = 3, respectively. Each vertical line represents one individual, the black lines separate samples from different localities, and the colors represent the different genetic clusters. The PUUV phylogenetic relationships are based on the concatenated S, M, and L segment sequences.

F-statistics and Bayesian clustering revealed consistent genetic subdivisions among vole sampling localities, which were however not consistent with geographical distances. Overall population differentiation was relatively low but statistically significant (*F*_ST_ = 0.027; *P* < 0.001), and also all pairwise comparisons among the sampling localities were significantly different from zero (*F*_ST_ between 0.014 and 0.047; all *P* < 0.001; Table S4). Clustering analyses with Structure applying the decision rule of Evanno et al. (2005) for the most likely *K* indicated the existence of two major genetic clusters in all voles sampled (Fig.7). The vast majority of individuals from Schledehausen were assigned to one cluster, and almost all voles from the other four sites were likely to belong to the second cluster.

The membership coefficients (*q*) of the individuals for the clusters showed very little variation across the ten Structure runs with *K* = 2, with an average standard deviation of 0.0012 (maximum 0.011). PUUV-infected individuals with *q* < 0.9 for the local population (*n* = 28) were always infected with a strain from the local virus cluster, indicating that PUUV transmission must have occurred locally if any of these voles were immigrants.

The inferred population structures of PUUV and bank voles did not completely correspond (Fig.7). The main subdivision in the bank voles separated Schledehausen from all other populations, which is in contrast to the topology of subdivision in PUUV populations. We performed additional Structure analyses with *K* = 3, which could potentially match the level of subdivision in PUUV (note that *K* = 3 had a lower likelihood than *K* = 2). The forced separation of the bank voles into three genetic clusters still did not match the subdivisions in the viruses, as individuals from Schledehausen were mostly assigned to one cluster, the majority of voles from Astrup to a second cluster, and some individuals from Ellerbeck were assigned to the Astrup cluster but most joined the third cluster with Bramsche genotypes (Fig.7).

## Discussion

This study provides a first comprehensive investigation of the spatial and temporal dynamics of PUUV in local populations together with its specific reservoir host. We detected genetic subdivisions in the bank vole and the PUUV populations at a very small geographical scale, but the genetic breaks were neither directly associated between host and virus, nor with geographical distances. Our persistence analyses revealed high turnover rates but also the presence of several virus types in the populations during multiple years of the study. PUUV prevalence in local vole populations and the incidence of human infections in the larger area did not follow tightly correlated temporal dynamics.

### Vole and PUUV population structure

The fragmented and often very region-specific PUUV outbreaks in central Europe have triggered a series of hypotheses about the transmission mechanisms of PUUV and the interactions with bank vole ecology and population dynamics (Schwarz et al. 2009; Tersago et al. 2009). Phylogeographic analyses of bank vole mtDNA have shown the presence of a single evolutionary lineage in most of Germany without finer geographical patterns within (Wójcik et al. 2010; Mertens et al. 2011). Some population-based analyses have resolved genetic structuring of bank voles at larger geographical scales (Rikalainen et al. 2012; White et al. 2013). The extent of genetic differentiation between the vole populations analyzed here was low but notable given the very short distances between the localities. There are no obvious barriers to dispersal such as rivers or major roads which might restrict gene flow specifically (Gerlach and Musolf 2000; Landguth et al. 2010 for general considerations), maybe except for the Schledehausen village, situated partially between the Schledehausen, Astrup, and Ellerbeck sites (Fig.1). To examine whether the separation of the Schledehausen population could go back to much earlier events, we analyzed the mitochondrial D-loop region of several samples from each of the vole populations but detected only very low genetic variation and no evidence of differentiation between the populations (results not shown; for methods, see Heckel et al. 2005; Mertens et al. 2011).

Higher genetic differentiation between the PUUV populations compared to the voles is consistent with the high evolutionary rates in these RNA viruses (Ramsden et al. 2008; Plyusnin and Sironen 2014; Zhang and Holmes 2014) and a lower effective size of their haploid genomes compared to the diploid hosts (Charlesworth 2009). No other study has specifically tested for population structure in PUUV, but a series of investigations detected the presence of different virus types at different localities (Sironen et al. 2001; Johansson et al. 2008; Razzauti et al. 2009). Beyond that, few studies have directly connected the investigation of genetic population structures in natural hosts and fast evolving viruses. For example, Torres-Pérez et al. (2011) found slightly more phylogeographic structure across Chile in Andes hantavirus than in its rodent host. Biek et al. (2006) showed much weaker structuring in North American cougars than in the feline immunodeficiency viruses carried by them. Thus, stronger genetic structuring among populations of fast evolving viruses than in their hosts may be more frequent in terrestrial systems but may not extend to systems with highly mobile hosts or vectors (Spackman et al. 2005; Chen and Holmes 2009; Liu et al. 2011, 2012). In our system, the mismatch between the deepest levels of host and virus population structure (Fig.7) may be related to, for example, a recent local replacement by a more divergent PUUV strain, but it will be necessary to extend our sampling to a wider area to test such a demographic scenario specifically.

It is unclear to which extent selection may contribute to differences between the virus populations, but the local scale of our analyses makes the contribution of ecological factors (e.g., habitat differences) rather unlikely. Differences between the bank voles as the actual environment in which PUUV replicates cannot be excluded given significant population structure, but nonsynonymous differences between the major geographical clades were not larger than variation within them (Fig.3). In additional consideration of the low population sizes of bank voles in some periods (Fig.2), genetic drift is thus likely to contribute strongly to differentiation between PUUV populations at the geographical scale investigated here.

### Temporal dynamics of PUUV

Our results demonstrate high PUUV turnover in local bank vole populations but also the persistence of some common sequence types through several years. The geographically consistent clustering of PUUV sequences (Fig.3) suggests that *de novo* mutations are a more likely source of the new and often transient sequence types than transmission from vole immigrants. Potential immigrants, that is, infected voles with assignment values <0.9 in the Structure analyses, always carried a PUUV type found at the site where they were trapped. Our sampling scheme is not suitable to examine vole dispersal patterns properly (see discussion in Schweizer et al. 2007), but if some of these individuals were immigrants, infection must have happened in all cases after the individual entered the local population.

The high evolutionary rates of PUUV support also a contribution of *de novo* generated variation to local strain diversity over the course of the study. Based on our substitution rate estimates and the concordant ones from Ramsden et al. (2008; but see Plyusnin and Sironen 2014; Zhang and Holmes 2014), we would expect on average about one new substitution per sequenced fragment over the eight years of the study. As mutation and substitution rate estimates may differ strongly between different study systems and show large variation across the genome (Sironen et al. 2001; Nemirov et al. 2010; Plyusnin and Sironen 2014; Zhang and Holmes 2014; Sheikh Ali et al. 2015), a more precise estimation of the contribution of *de novo* variation to temporal variation in PUUV population diversity will have to await dedicated analyses.

Rapid temporal turnover in RNA viruses is often seen as a consequence of their high evolutionary rates and fast response to selection (Holmes 2009; Łuksza and Lässig 2014). Fitness differences between strains have been suggested for PUUV (Sironen et al. 2008) and other viruses (Bull and Molineux 2008; Alto et al. 2013), yet our phylogenetic analyses did not reveal the temporal clustering often seen in other RNA viruses [e.g., influenza A virus (Łuksza and Lässig 2014); dengue virus (Twiddy et al. 2002; Bennett et al. 2003)]. Such a topology is normally caused by strong selection in which only very few strains with higher fitness give rise to new strains in the next time period. The absence of such topologies here (see Fig.4B,C and Figure S1) may suggest that a period of eight years is not long enough in PUUV evolution and/or that selection was not sufficiently strong. Indeed, PUUV is often seen as causing only a minor reduction of the fitness of infected reservoir hosts (but see Kallio et al. 2007; Vaheri et al. 2013b). However, given relatively low local effective population sizes of PUUV (Fig.6) associated with low population sizes of bank voles in winter (Kallio et al. 2007), it is parsimonious to assume an important role of genetic drift for PUUV strains in natural populations, and thus, differences in persistence periods in the vole populations may not be related to any fitness differences between strains. Our analyses of partial PUUV genomes likely missed genetic variation in these strains, but potential additional standing variation or the accumulation of *de novo* mutations in other genome regions of the persisting strains cannot erase the global patterns of persistence detected here. Obviously, full genome data combined with experimental approaches would be favorable to assess fitness differences between PUUV strains in detail. Additionally, a further extended sampling of PUUV – over a longer period of time and with multiple sampling times per year – would certainly deepen our understanding of the viral population dynamics in the future.

Reassortment often contributes to genetic variation in RNA viruses with segmented genomes, for example, in influenza virus (Nelson and Holmes 2007; Łuksza and Lässig 2014) and bluetongue virus (Coetzee et al. 2012). Natural reassortants were described for the Sin Nombre hantavirus (Henderson et al. 1995), and Razzauti et al. (2013) reported 19% reassortant viruses in regional PUUV populations in central Finland. In the present study, there was no evidence of reassortment between the PUUV segments or for multiple infection of a vole. Virus strains in our study populations are genetically closer to each other than in some of the Finnish populations (Razzauti et al. 2013), which might reduce the probability of detecting reassortment events. The absence of any evidence of reassortment in our German populations over eight years, though, raises the question whether the frequency of multiple infections and/or reassortments differs between populations or geographical regions stochastically or whether this is related to environmental factors. At present, it appears that northern European PUUV strains might have higher evolutionary potential than central European ones because reassortment has been reported more often from northerly regions (Plyusnin et al. 1997; Razzauti et al. 2008, 2013). However, the more frequent use of sequence data in Fennoscandia may bias the reports of reassortants in the literature. Thus, it remains to be tested whether the evolutionary avenues of PUUV in Europe might differ even more with geography than previously anticipated.

## Conclusion

This study provides a first bottom-up perspective of the population dynamics of PUUV and its natural host, the bank vole, at a very fine geographical scale. Our temporal analyses indicate limited cofluctuation of PUUV prevalence in local natural host populations or host abundance and outbreaks in the surrounding human population (Fig.2; but see, Olsson et al. 2003; Kallio et al. 2009; Tersago et al. 2009, 2011). The marked substructure among PUUV from different localities, together with the high level of variability and the appearance and disappearance of apparently only slightly different sequence types in local vole populations, offers the opportunity to develop strain distribution maps. PUUV sequence information from human infections could then be used to identify at a relatively fine scale the geographical region where and potentially under which circumstances the transmission of the virus to the patient occurred. Coupling investigations of natural host populations with molecular data from human patients would thus open new possibilities for an epidemiological understanding of PUUV, and potentially for ‘uncovering the mysteries of hantavirus infections’ (Vaheri et al. 2013b) in general.

## Data archiving statement

Earlier sequence data are available on GenBank under the accession numbers JN696358, JN696364, JN696355, DQ518217, and KJ994776-KJ994778, and new sequence data under KR047195-KR047313 (S segment), KR047314-KR047431 (M segment), and KR04743-KR047554 (L segment). Microsatellite genotypes and location data are available in Dryad number doi:10.5061/dryad.p1k7k and phylogenetic data in TreeBASE http://purl.org/phylo/treebase/phylows/study/TB2:S17360.

## Supporting Information

**Figure S1.** Neighbor-joining phylogenetic tree inferred from concatenated S, M and L Puumala virus (PUUV) sequences with strain Sotkamo as an outgroup. **Figure S2.** Persistence of Puumala virus types in the Astrup vole population for each genome segment and for the three segments concatenated. **Table S1.** Primers used for RT-PCR amplification and sequencing of Puumala virus S, M and L segments. **Table S2.** Pairwise *F*_ST_ values (lower diagonal) and *P*-values (upper diagonal) for the Puumala virus populations at five localities. **Table S3.** Pairwise *F*_ST_ values (lower diagonal) and P-values (upper diagonal) for the Puumala virus populations Schledehausen (Schle) and Astrup. **Table S4.** Pairwise *F*_ST_ values (lower diagonal) and *P*-values (upper diagonal) for the bank vole populations at five localities.
