# Supplementary material for: Spatiotemporal dynamics of Puumala hantavirus associated with its rodent host, Myodes glareolus
Source: Evol Appl. 2015 May 29;8(6):545–59. doi: 10.1111/eva.12263 (PMC4479511; doi:10.1111/eva.12263)
Supplement: Supplementary file 1 [file eva0008-0545-sd1.docx]

**Supplementary material**

**Table S1 Primers used for RT-PCR amplification and sequencing of Puumala virus S, M and L segments**

| Segment | Primer name | Sequence 5’ -3’ | Reference |
| --- | --- | --- | --- |
| S | 342fw | TAT GGT AAT GTC CTT GAT GT | Essbauer et al. (2006) |
|  | 1102rev | GCC ATD ATD GTR TTY CTC AT | Essbauer et al. (2006) |
|  | 334fw | TAT GGA AAT GTC CT T GAT GT | this paper |
|  | 1065rev | GCC ATA ATA GTA TTT CTC ATG | this paper |
|  | PUUV390fw | GGN CAR ACA GCR GAT TGG T | Essbauer et al. (2006, nested primer) |
|  | cPUUV721rev | ACH CCC ATN ACW GGR CTY AT | Essbauer et al. (2006, nested primer) |
|  | 508fw | CGT ATT CGG TTT AAG GAT GAT | this paper |
|  | 508rev | GTA TCA TCC TTA AAC CGA ATA | this paper |
| M | C1fw | CCC CCT GAT TGT CCT GGT GTA G | Plyusnin et al. (1997) |
|  | C2rev | CCA ACT CCT GAA CCC CAT GC | Plyusnin et al. (1997) |
|  | C1mfw | CCA GCT GAT TGC CCA GGG GTA G | this paper |
|  | C2mrev | CCT ACT CCT GAG CCC CAT GC | this paper |
|  | M3mfw | CTT TGA GAT ATA CTA GGA AG | Pilaski et al. (1994) |
|  | M4rev | GTT GTT GCA AAT GCA CAT | Pilaski et al. (1994) |
|  | 2620fw | GGT CCA CTT GAA CAA GGC GG | this paper |
|  | 2620rev | GTC CGC CTT GTT CAA GTG GAC C | this paper |
| L | HanLF1 | ATG TAY GTB AGT GCW GAT GC | Klempa et al. (2006) |
|  | HanLR1 | AAC CAD TCW GTY CCR TCA TC | Klempa et al. (2006) |
|  | HanLF2 | TGC WGA TGC HAC HAA RTG GTC | Klempa et al. (2006) |
|  | HanLR2 | GCR TCR TCW GAR TGR TGD GCA A | Klempa et al. (2006) |
|  | 2935fw | ATG TAT GTA AGT GCA GAT GC | this paper |
|  | 3367rev | AAC CAG TCT GTT CCG TCA T | this paper |

**Table S2** **Pairwise F_ST_ values (lower diagonal) and P-values (upper diagonal) for the Puumala virus populations at five localities.**

|  | Schledehausen | Astrup | Ellerbeck | B. Varus | B. Tower |
| --- | --- | --- | --- | --- | --- |
| Schledehausen |  | <0.0001 | <0.0001 | <0.0001 | 0.99 |
| Astrup | 0.25 |  | 0.0002 | <0.0001 | 0.99 |
| Ellerbeck | 0.24 | 0.18 |  | 0.0011 | 0.99 |
| B. Varus | 0.36 | 0.34 | 0.35 |  | 0.99 |
| B. Tower | 0.27 | 0.23 | 0.20 | 0.6 |  |

**Table S3** **Pairwise F_ST_ values (lower diagonal) and P-values (upper diagonal) for the Puumala virus populations Schledehausen (Schle) and Astrup.** The comparisons are between samples from different years but the same site.

| Schle | 2005 | 2007 | 2008 | 2009 | 2010 | 2011 | 2012 |
| --- | --- | --- | --- | --- | --- | --- | --- |
| 2005 |  | 0.4882 | 0.9990 | 0.5097 | 0.2255 | 0.2509 | 0.2382 |
| 2007 | 0.30 |  | 0.1709 | 0.999 | 0.4179 | 0.0390 | 0.0009 |
| 2008 | 0.00 | 0.16 |  | 0.1474 | 0.038 | 0.0283 | 0.0087 |
| 2009 | 0.33 | -0.14 | 0.14 |  | 0.7275 | 0.3789 | 0.0488 |
| 2010 | 0.20 | 0.01 | 0.13 | -0.07 |  | 0.0175 | 0.0000 |
| 2011 | 1.00 | 0.56 | 0.42 | 0.50 | 0.34 |  | 0.9990 |
| 2012 | 0.61 | 0.50 | 0.43 | 0.34 | 0.33 | -0.12 |  |

| Astrup | 2007 | 2008 | 2009 | 2010 | 2011 | 2012 |
| --- | --- | --- | --- | --- | --- | --- |
| 2007 |  | 0.0058 | 0.999 | 0 | 0.0293 | 0.0009 |
| 2008 | 0.43 |  | 0.999 | 0.58 | 0.039 | 0.4482 |
| 2009 | 0.58 | 0.30 |  | 0.4082 | 0.3378 | 0.0908 |
| 2010 | 0.45 | -0.005 | 0.23 |  | 0 | 0.1005 |
| 2011 | 0.65 | 0.48 | 1.00 | 0.51 |  | 0.121 |
| 2012 | 0.41 | 0.008 | 0.31 | 0.08 | 0.32 |  |

**Table S4 Pairwise F_ST_ values (lower diagonal) and P-values (upper diagonal) for the bank vole populations at five localities.**

|  | Schledehausen | B. Tower | B. Varus | Astrup | Ellerbeck |
| --- | --- | --- | --- | --- | --- |
| Schledehausen |  | <0.0001 | <0.0001 | <0.0001 | <0.0001 |
| B. Tower | 0.04704 |  | 0.0413 | 0.0001 | <0.0001 |
| B. Varus | 0.04098 | 0.01575 |  | <0.0001 | <0.0001 |
| Astrup | 0.02720 | 0.03275 | 0.02872 |  | <0.0001 |
| Ellerbeck | 0.01851 | 0.03113 | 0.03389 | 0.01406 |  |


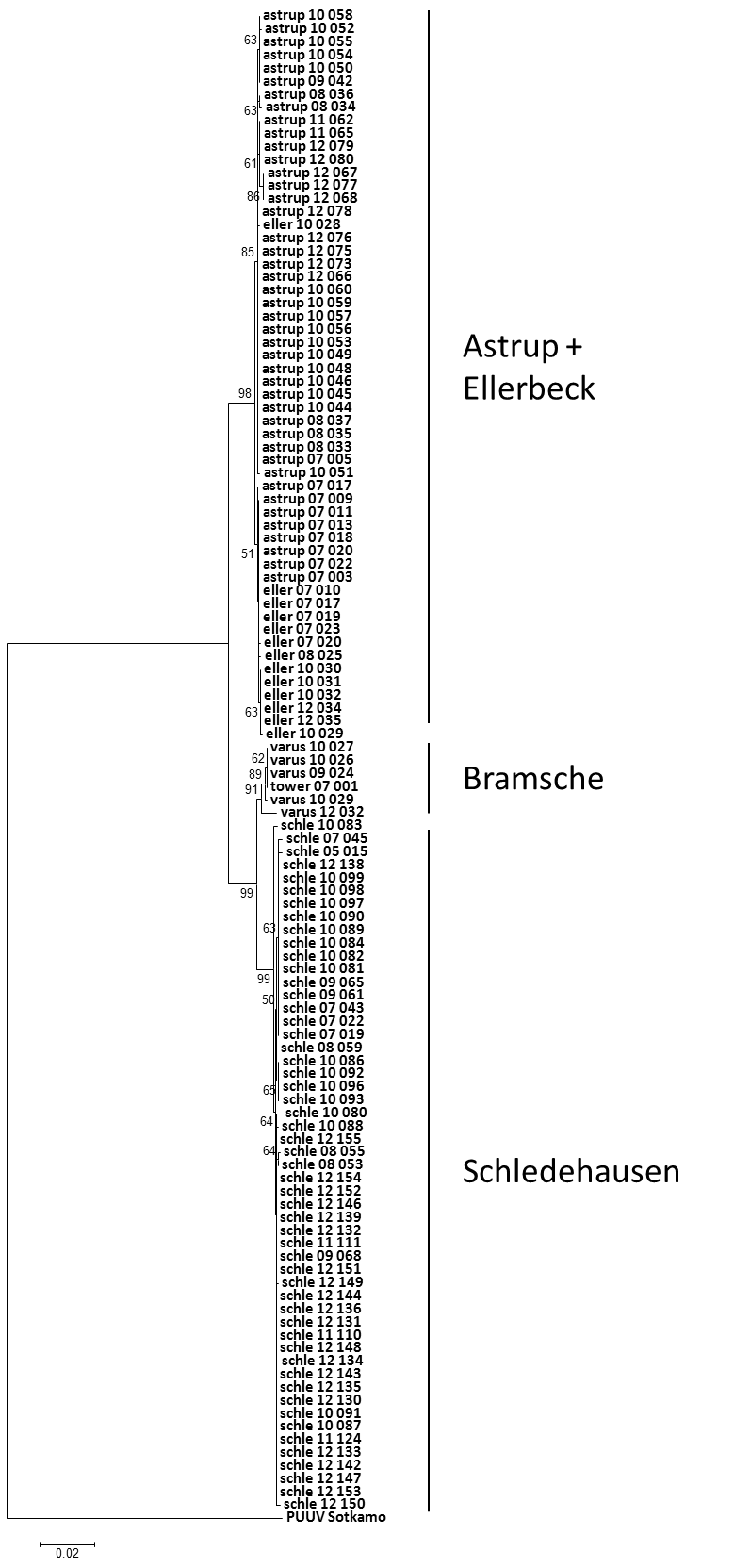


**Figure S1 Neighbor-joining phylogenetic tree inferred from concatenated S, M and L Puumala virus (PUUV) sequences with strain Sotkamo as an outgroup**. Sequence names indicate the geographical origin of the sample (schle, Schledehausen; astrup, Astrup; eller, Ellerbeck; varus, Bramsche Varus; tower, Bramsche Tower), followed by two digits indicating the sampling year. Bootstrap values are given if they exceeded 50%.


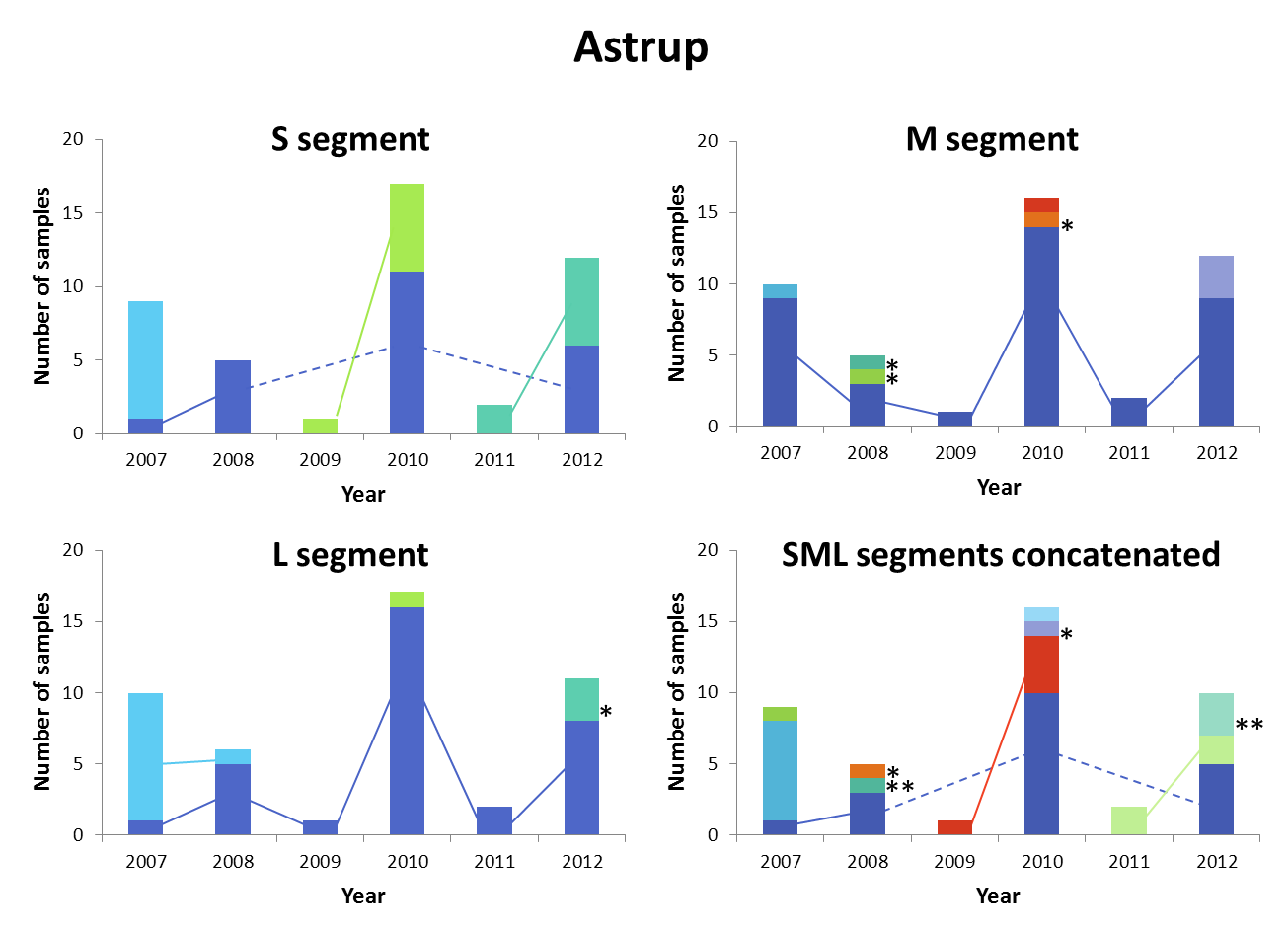


**Figure S2** **Persistence of Puumala virus types in the Astrup vole population for each genome segment and for the three segments concatenated**. The colors indicate different virus sequence types, and the lines connect the same virus type in different years. Asterisks indicate virus types that present non-synonymous substitutions.
